# The ideal of biopsychosocial chronic care: How to make it real? A qualitative study among Dutch stakeholders

**DOI:** 10.1186/1471-2296-13-14

**Published:** 2012-03-12

**Authors:** Anneke van Dijk-de Vries, Albine Moser, Vera-Christina Mertens, Jikke van der Linden, Trudy van der Weijden, Jacques Th. M van Eijk

**Affiliations:** 1 Department of General Practice, Maastricht University, School for Public Health and Primary Care (CAPHRI), PO Box 616, 6200 MD Maastricht, the Netherlands; 2 Department of Social Medicine, Maastricht University, School for Public Health and Primary Care (CAPHRI), Maastricht, the Netherlands; 3 Faculty of Care and Nursing, Zuyd University, Heerlen, the Netherlands

## Abstract

**Background:**

Chronically ill patients often experience psychosocial problems in everyday life. A biopsychosocial approach is considered to be essential in chronic care. In Dutch primary health care the current biomedically oriented clinical practice may conflict with the biopsychosocial approach. This study is aimed to explore the views of Dutch stakeholders on achieving a biopsychosocial approach to the care of patients with chronic diseases.

**Methods:**

In a qualitative explorative study design, we held semi-structured interviews with stakeholders, face-to-face or by telephone. Data were analysed using content analysis. Thirty representatives of Dutch patients with chronic illnesses, primary care professionals, policy makers, health inspectorate, health insurers, educational institutes and researchers were interviewed.

**Results:**

Stakeholders were aware that a systematic biopsychosocial care approach is lacking in current practice. Opportunities for effective change are multidimensional. Achieving a biopsychosocial approach to care relates to active patient participation, the training of professionals, high-quality guidelines, protocols and tools, integrated primary care, research and financial issues.

**Conclusions:**

Although the principles and importance of the biopsychosocial model have been recognized, the provision of care that starts from the medical, emotional or social needs of individual patients does not fit in easily with the current Dutch health care system. All parties involved need to make a commitment to realize the ideal of biopsychosocial chronic care. Together they need to equip health professionals with skills to understand patients' multifaceted needs and to reward integrated biopsychosocial care. Patients need to be empowered to be active partners in their own care.

## Background

Like that in other Western countries, Dutch primary health care is being challenged by the rapidly rising prevalence of chronic diseases [1,2]. Given the long-term nature of chronic conditions, there is a growing recognition that patients need to be supported in managing their own health [3]. Good self-management skills are associated with improved patient-reported outcomes and reduced health care costs [4]. Lorig distinguishes three self-management tasks: medical, emotional and role tasks [4]. Ideally, health professionals should systematically and simultaneously address the way patients cope with these tasks. This would require them to apply the biopsychosocial model, rather than using a narrow biomedical focus on patient care [5].

Although the biopsychosocial care approach is considered to be essential for patients with chronic disease, it appears to be poorly embedded in the Dutch primary care system. In the last decade, chronic care for patients with type 2 diabetes, COPD and cardiovascular disease has been largely transferred from general practitioners (GPs) to practice nurses (PNs). The indicators describing the GPs' and PNs' performance are only defined in terms of biomedically oriented clinical guidelines and standards of quality of care [6,7]. The reimbursement of care expenditures by insurance companies, which is now regulated on the basis of 'diagnosis treatment combinations' (DTC, a Dutch variant of Diagnosis Related Groups) is also based on these guidelines and care standards [8]. Consequently, most interventions are defined in terms of control of biomedical aspects, like glycaemic control in diabetes patients, ignoring the psychosocial impact of chronic disease [4,8,9]. It is not surprising, therefore, that chronic patients with psychosocial problems often receive only biomedically oriented, and thus incomplete, treatment [10,11].

In the Dutch health care system, mental health care is provided in both primary health care (by GPs, specialized nurses in general practice, primary care psychologists and psychotherapists) and secondary health care. For patients with a physical chronic disease it is relevant that biomedical and psychosocial care are provided in an integrated instead of separated way, since they often encounter psychosocial problems that may hinder their ability to manage their disease [12]. In this study, we seek to gain more insight in the needs and obstacles for the implementation of biopsychosocial chronic care. Therefore, we examined the views of all who have a stake in realizing a Dutch biopsychosocial care model for chronic patients. These stakeholders were representatives of patients, primary care professionals, health policy makers and inspectorate, health insurers, educational institutes and research. The research questions were:

1. What are the views of stakeholders in Dutch chronic care on the current state of biopsychosocial care?

2. Which barriers and facilitators do stakeholders perceive as regards the implementation of biopsychosocial chronic care?

## Methods

A qualitative explorative design was used to get a bottom-up overview of stakeholders' views regarding the biopsychosocial care of people with chronic diseases.

### Participant recruitment

Stakeholders who were knowledgeable and/or had experience of the topic in our study were invited to participate. To obtain richly detailed data, we selected participants using purposive sampling and snowball sampling [13], ensuring that all groups of stakeholders were included to get a diversity of perspectives: patients, primary care professionals such as GPs and PNs, health policy makers and inspectors, health insurers, health education professionals, and researchers. Using the purposive sample method we made a list of relevant Dutch institutions with regard to the topic of our study. On their web pages we searched for contact details. We also used our network to approach principal investigators in a specific research area related to the subject of our study. Using the snowball sampling method we asked participants to recommend others that could give valuable input to the study.

Experts were approached by telephone for a short introduction of the study and were then sent a letter with more information on the study. Participation was voluntary. Anonymity and confidentiality was ensured. If experts were willing to participate, an appointment was arranged. Thirty stakeholders were invited. Nobody refused to participate. Verbal informed consent was obtained. This consent was audio recorded.

### Data collection

Nine face-to-face and 21 telephone interviews were held between December 2009 and June 2010, based on an open-ended, semi-structured interview guide (see Additional file 1). The interview guide was partly adapted to fit the respondents' background or on the basis of findings of prior interviews. The interviews lasted 16 to 59 minutes. The interviewers (AND, VM, JL) started with face-to-face interviews. In view of the distances to be travelled, they then continued with telephone interviews, since face-to-face contact with respondents and immersion in their environment was not considered necessary to receive the relevant information about the subject of our study. Telephone interviews have been reported to be a valid data collection tool [14]. Signals like hesitations and pauses were used as cues to ask probing questions. All interviews were recorded. As regards transcriptions in qualitative research, Strauss and Corbin [15] recommended: "The general rule of the thumb here is to transcribe only as much as is needed (...) The actual transcribing should be selective" (p. 30). In our procedure, the interviewer who performed a certain interview listened to the audiotape and made a comprehensive summary of the complete interview. Summaries were structured according to the interview guide. All statements that were more or less related to the research questions were transcribed verbatim and added to the summaries. The researcher who did the analysis (AM) listened to all audio taped interviews again to check the accuracy of the interview summaries. During this phase any potentially significant statements that were missed in the first transcriptions were also transcribed verbatim. This was cross-checked with the interviewers. The summaries were discussed during analytical sessions.

#### Analysis

A content analysis was performed, based on the constant comparative method as described in Grounded Theory [15]. We used the constant comparative method as an analytical tool. Codes and categories relating to biopsychosocial care emerged inductively from the interview data and deductively from the constant comparison. The researcher who did the analysis (AM) first read the interview summaries and listened to the audio-taped. Next, relevant sections of the interview data were coded by AM, using codes that were often based on descriptions used by respondents. These codes were grouped into categories and subcategories which best characterized the data collected. Throughout the analysis, the codes and categories were constantly compared and contrasted within and among the interviews. Memos [15] were written throughout the process. Data saturation [15] was reached after 14 interviews. The remaining interviews served to reinforce the data of the phenomenon under study and to fill the categories. It has enriched our material by collecting valuable quotes from the different stakeholders. Quotes are included in the results section to illustrate and amplify our findings.

### Validity

Several strategies were used to ensure credibility [16] (internal validity). Firstly, 63 persons who were knowledgeable and/or had experience in chronic care (including all interviewees) were invited for an invitational conference, at which we presented them with the first draft of our results as input for further dialogue. The conference was attended by 34 participants (including 13 of the interviewees). During the conference, field notes were made. The conference turned out to support our study results as no new perspectives arose regarding the needs and opportunities to achieve a biopsychosocial care approach. With regard to the interviews and analysis process, research team meetings were held to fine-tune the activities between the various interviewers [17] and researchers. They met frequently to reflect upon the interview guide, sampling and summaries of the interviews, as well as on the analysis process, codes, and categories and subcategories that emerged. A member check was performed by submitting each interview summary to the respondent for approval.

## Results

The sample consisted of 30 stakeholders (18 men, 12 women) from various backgrounds. Most were involved in two (n = 12) or three (n = 3) domains. Patients were represented by three persons with a chronic illness and two board members of a national patient federation (one of whom had recently moved to a professional association for PNs). The sample included six GPs and three PNs. Mental health care was represented by two participants involved in professional associations of primary care mental health workers and one psychiatrist. Three participants were involved in medical education (training GPs and PNs). Three participants were involved in the development of national guidelines. The sample also included two health insurers and two health inspectors. Four participants were involved in national health policy. Researchers (n = 11) included principal investigators and others involved in research on primary care, chronic care, mental health care, implementation science, and general practice. Most of them were also professionally involved in health care (n = 4), guideline development (n = 2), mental care (n = 1), national health policy (n = 1) or medical education (n = 2). Our data extraction was confirmed in the member checks.

### Achieving a biopsychosocial approach to chronic care

Respondents underlined the impact of chronic conditions on psychosocial functioning and patients' needs for support in dealing with a chronic disease in everyday life. Although they considered a biopsychosocial approach to be inherent in being a primary care professional, respondents confirmed the current lack of a systematic approach in providing psychosocial care to patients with a chronic disease. The present use of the biopsychosocial model mainly depends on the individual skills of the professionals rather than on a well-planned strategy. Respondents perceived a need for simultaneous changes at various levels.

#### Recognition of psychosocial problems: a shared responsibility

Primary care professionals need to be aware that psychosocial care is an important adjunct to medical care. Both patients and professionals argued that it is difficult to determine when or whether health professionals should be involved, as psychosocial problems were perceived as belonging to the normal life of chronically ill people. Hence, neither doctors nor patients do sufficiently address psychosocial problems.

*'What is the dividing line between a 'normal reaction' and a 'pathological reaction'? When does a mood problem turn into a depression? When is gloominess no longer acceptable?' *[researcher]

Respondents attached great importance to providing care that starts from the perceived, multifaceted needs of patients. In this regard, interviewees emphasized that patients and their social environment need to play an active role in the whole care process. Their participation is crucial for the early recognition of psychosocial problems and for discussing them with a doctor during consultations.

*'The patients themselves and their social environment obviously play an important role as well. The patient has to notice that this is more than just their chronic somatic disorder, that there is something wrong with their attitude, their situation, their mood. ... This care environment may also include the patient's relatives.' *[researcher, health policy maker]

Some wondered how a health professional assesses patients' needs, or to what extent patients themselves define their needs. One patient noted that many (particularly older) patients are passive recipients of care, who look up to a health professional as an authority. Patients' expectations about psychosocial care in general practice seemed to be low, as they think health professionals do not have time to address this kind of problem or cannot empathize with problems the way fellow patients can.

#### Training of health professionals

Most respondents considered training to be a major facilitating factor to ensure that the biopsychosocial model becomes integrated in chronic care. Respondents representing educational institutes confirmed their intention to train their students to develop a joint focus on the biological, psychological and social dimensions of illness. However, they argued that teaching programmes should make a greater effort to teach students to start from the patient as a person rather than the disease.

*'Right from the start of the courses in year 1, students should think in terms of patients and care needs. So the teaching courses really need to be changed. Doctors should not be trained so much to think in terms of diseases, but to think in terms of care needs.' *[professional involved in research, teaching and guideline development]

Primary care professionals (especially GPs and PNs) should be sensitized to identify psychosocial needs associated with medical health problems, and care providers in chronic care also need to be trained to work in multidisciplinary teams. Other issues that were mentioned included more training in communicative competencies like listening or recognizing nonverbal signals, and training to monitor patients' perceived problems in daily functioning.

#### Guidelines, protocols and tools

Current guidelines for chronic care do not adequately integrate medical and psychosocial aspects, or multimorbidity. Multimorbidity can relate to multiple somatic diseases but also to psychosocial problems. Respondents mentioned the complicated task of incorporating and presenting all these aspects in conveniently structured guidelines. Some suggested allowing more room for the patient's perspective in guidelines, by involving patients in the guideline development process.

Quality assurance indicators, preferably derived from clinical practice guidelines, were also mentioned. To date, outcome parameters have been dominated by biomedical indicators such as HbA1c (blood sugar level). The public health quality indicators defined by the Dutch Health Inspectorate also include only biomedical parameters. Respondents underlined the need for process and outcome quality indicators for psychosocial care.

*There is no interest in my patients' quality of life, which is what should really be the performance measure. In practice, your performance is judged on the basis of very simple outcome measures - × number of decimals of the HbA1c values*. [GP, researcher]

The majority of respondents said hat GPs and PNs needed additional tools such as questionnaires to help them with signalling, diagnosing and selecting interventions, as well as monitoring and evaluating biopsychosocial care. Several respondents mentioned systematic screening to identify patients with a need for psychosocial care, while at the same time being cautious about the downside, i.e. the generation of false-positive screening results. Some suggested a stepwise method of case-finding to differentiate between mild, moderate and serious problems and to provide care that fits the patient's needs. In addition to tools to detect specific issues (like social isolation or poly-pharmacy), respondents asked for communication techniques or less laborious assessment instruments to identify individual patients' needs.

*'My ideal, if we could start again, would be that I should diagnose, for instance, a diabetes patient, that of course we offer all the options that science has to offer, but that in addition to that we'd have a kind of list, 10 to 20 points, that you take the patient through to see what their problems are in everyday life. ... Using the doctor's experience to list current problems and expectations and then check the same list every year or so to see if you are still working on the same goals.' *[GP, researcher]

#### Integrated primary care

Respondents perceived an integrated primary care system as one of the cornerstones of biopsychosocial care, involving clearly defined pathways and effective teamwork among all caregivers in primary and secondary care.

*What we need is a closely integrated first-line health care system, with clear lines. Everyone involved should know their own place in the collaborative model.' *[GP, researcher]

Disease management programmes that include prevention, monitoring and treatment might already represent a step forward to integrated chronic care. However, some caution is needed as these programmes are still driven by diseases rather than by patient needs.

Some pointed out that integrated primary care starts within the small-scale setting of a GP practice. Delegation of tasks from GPs to PNs, practice assistants and/or nurse practitioners, and the variety of responsibilities and specialities makes effective teamwork very complex. In addition to this, there are several kinds of mental health care professionals involved in Dutch primary care. This highlights the need for a clear division of tasks, responsibilities and communication processes among the various health professionals.

*'A sort of step-by-step plan: what aspects should be the responsibility of the GP, what should be done by a psychiatric nurse? As a practice nurse, you need to be aware of your limitations. There comes a point where I have to say: this is beyond my professional competence, and should be dealt with by the practice psychiatric nurse.' *[Health care professional]

Some emphasized that GPs should remain primarily responsible for the patient's care process. Others mentioned that PNs are increasingly taking on a coordinating case-manager role for chronic patients. A concern was that PNs who provide care for patients with a physical chronic disease may not correctly identify and treat psychosocial problems, since they have little experience with psychological and social health problems.

#### Financial issues

The professionals mostly mentioned a lack of time to incorporate psychosocial care in their routine practice. Time constraints were related to the available consultation time, workload and financial constraints. In this regard, respondents commented on the payment system for primary care. Some emphasized the potential benefits of the Dutch reimbursement system, which offers all-inclusive payment for people with chronic conditions to a multidisciplinary team. This stimulates the delivery of efficient and integrated chronic care. However, the emphasis on diagnosis-treatment combinations (DTCs) rather than on aspects like comorbidity or multimorbidity does not encourage professionals to start from the multifaceted biopsychosocial needs of individual patients.

*'I'm not against DTCs at all; I think they're the best way to work in an output-driven manner. I really think they are necessary in health care. But you may wonder whether the current DTC concepts are useful. I could imagine that I might see a patient and think I will define a DTC not with the aim of treating the disease but actually treating the patient. Looking at the patient in a holistic manner. That's the right way.' *[professional involved in research, teaching and guideline development]

Respondents argued that psychosocial care should be a general module within all DTCs. This would mean making it an integrated part of all national care standards on which health insurers base their payments. If no reimbursement is given, professionals in the field will not systematically include this aspect in their daily care.

#### Research

The majority of respondents mentioned research as an essential prerequisite for achieving a biopsychosocial approach to care. If interventions are to be incorporated in care standards and hence included in the reimbursement fees, they have to be evidence-based. The development of evidence-based biopsychosocial self-management interventions needs to be given greater priority on the research agenda of major funding organisations. Respondents preferred intervention research with patient-reported outcomes such as quality of care, patient satisfaction and healthcare consumption. However, this kind of research is complex among patients with comorbidity or multimorbidity, and patients with multifaceted health problems are often excluded. Respondents also suggested more research with the aim of improving integrated primary care. In addition, they stressed the importance of research into the patients' needs.

## Discussion

Our results show that the biopsychosocial model in chronic care is an ideal shared by the respondents, and the interview findings were confirmed by the invitational conference. Treating patients as whole persons in their social and personal context implies that health professionals should take time to detect and address psychosocial problems in patients with a physical chronic disease. The stakeholders in our study agreed that this is insufficiently happening in current practice.

Our findings provide a sound illustration of the complex shared responsibility of stakeholders to overcome the barriers regarding a biopsychosocial approach in the care for patients with chronic disease. The study is not an in-depth analysis of specific stakeholder views. Furthermore, the study is limited as it is specific to the Dutch context.

Barriers to the use of the biopsychosocial model during medical encounters relate to care providers being inadequately equipped for this approach, resulting in both over-diagnosis and under-diagnosis of psychological problems [10]. Stakeholders in our study asked for tools to distinguish between normal responses to chronic illness and 'disorders' that need specialist care. Such discriminative tools can be helpful in guiding health professionals towards psychosocial aspects of being chronically ill, but when taken too literally, they can also create unnecessary boundaries [18]. Some have argued [18,19] that seeing symptoms in the light of what is going on in a persons' life might enhance biopsychosocial care. Rather than labelling patients according to psychiatric criteria, they allocate an important role to patients' own stories and watchful waiting. This asks for a paradigm shift towards a biopsychosocial self-management approach that really starts from the patients' perceived needs. Health professionals already have to be familiarized with this care approach during their professional training. In this regard, Dutch experts on care for the elderly [20] recently called for interprofessional training for health professionals, as effective communication and teamwork skills are required to implement biopsychosocial self-management by patients.

A biopsychosocial self-management approach requires health professionals who view patients as experts on their own lives and thus responsible for their own health. It also supposes that patients express their needs regarding their medical, emotional and role tasks. This is critical in patients with low health literacy and/or low socio-economic status, as they are at higher risk for distress and depression than their counterparts with higher socio-economic status, and are less able to use a pro-active coping style [21]. The expectation that relatively short consultations with primary care professionals can address complex biomedical and psychosocial problems in addition to the other tasks like forming partnerships, providing preventive care and coordinating care has been criticized, especially with regard to the socially disadvantaged [22]. Stretching consultation times and providing financial resources might not be the only solution. What is needed is nation-wide patient empowerment. The various patient societies in the Netherlands can use their networks and capacities to encourage individual patients to become more actively involved in their own care [23].

We believe that the current structure of Dutch health care funding impedes the introduction of biopsychosocial care. The shift from a primarily government-funded model to a regulated, quasi free-market model and the introduction of DTCs in primary care, offering 'all-inclusive' reimbursement for people with a chronic condition, have resulted in greater powerfor health insurers. Their interest in financial efficiency means that professionals are showered with documentation on 'hard' (biomedical) outcome parameters for administrative and billing purposes [24,25]. Furthermore, the disease-oriented DTCs may provoke fragmentation of care as they focus on a single chronic disease [8]. Guidelines and standards based on a biopsychosocial self-management approach (like the Dutch care standard on COPD [26]) can hardly prevent the problem of focusing on the disease rather than on the patient. DTCs should include general reimbursement categories such as care coordination and focusing attention on the way patients cope with the consequences of their chronic disease in everyday life.

Unexpected side-effects of the quasi free-market system are the niches for bottom-up societal initiatives such as small-scale community care [27]. The collective sense of responsibility for Patients' well-being that is found in small, autonomous care teams might facilitate biopsychosocial care. Further research is necessary to investigate the impact of such initiatives on patients' wellbeing and biopsychosocial self-management, as well as on health care costs.

There is no doubt about the negative impact of psychosocial problems on treatment compliance [28], deterioration of chronic conditions [29,30] and health care costs [31]. Empathic communication and raising positive expectations have a favourable influence on patient outcomes [32]. Hence, neglecting psychosocial aspects of chronic disease in health care is in direct conflict with scientific evidence. Health professionals need to wake up to this notion, and need to define their position in clinical guidelines and standards. This would allow biopsychosocial care to also become an integrated part of the reimbursement of care. Patients should be involved in the guideline development process to ensure that their values and biopsychosocial needs are incorporated in evidence-based guidelines [33].

To make biopsychosocial care real, a rational choice would be to start with further equipment of PNs as they monitor chronic patients on a regular basis. According to their professional profile, PNs are expected to obtain a reasonable level of competences in psychosocial care [34]. Our study has addressed the gap between what is written in this profile and what is done in daily practice. Together stakeholders need to equip PNs for providing biopsychosocial care.

## Conclusions

The current Dutch health care system does not encourage the provision of self-management support that starts from the medical, emotional and social needs of individual patients - often with more than one chronic condition. If we expect patients to be responsible for their own health and be active partners in care, empowerment needs to be given high priority on the agenda of patient organisations. Scientific research, educational institutes and guideline developers have to equip multidisciplinary health teams with skills, tools and guidelines that help them to provide care starting from the patients' needs. In addition, health policy makers, health insurers and the health inspectorate have the challenging task of developing a supportive health care and financial system in which health professionals are given incentives to provide biopsychosocial care. It is important that these key players take up their responsibility to realize the ideal of biopsychosocial chronic care.

## Abbreviations

COPD: Chronic obstructive pulmonary disease; GP: General practitioner; PN: Practice nurse; DTC: Diagnosis treatment combinations, Dutch variant of Diagnosis Related Groups.

## Competing interests

The authors declare that they have no competing interests.

## Authors' contributions

The manuscript was drafted by AM and AND. Both had full access to all interview data, took responsibility for the integrity of the data and the accuracy of the analysis. JThME wrote the proposal and was the project leader. He supervised the research project throughout. TW provided critical feedback on all drafts. VCM and JL carried out interviews and provided comments on the several drafts of the manuscript. All authors read and approved the final manuscript.

## Ethical approval

Ethical approval was not required according to the Dutch 'Medical Research Involving Human Subjects Act ('WMO') http://www.ccmo-online.nl/main.asp?pid=43&thid=57&catid=2#a1assessed 15.08.2011. Representatives of patients were not in actual need of psychosocial care. They were not interviewed about their own care but on chronic care in general.

## Pre-publication history

The pre-publication history for this paper can be accessed here:

http://www.biomedcentral.com/1471-2296/13/14/prepub

## Supplementary Material

Additional file 1 **Interview guide**. Click here for file
