# Supplementary material for: The ideal of biopsychosocial chronic care: How to make it real? A qualitative study among Dutch stakeholders
Source: BMC Fam Pract. 2012 Mar 12;13:14. doi: 10.1186/1471-2296-13-14 (PMC3355054; doi:10.1186/1471-2296-13-14)
Supplement: Additional file 1 — Interview guide. [file 1471-2296-13-14-S1.DOC]

**Appendix 1 Interview guide**

We provide the main questions. Depending on the stage of analysis and on the participants we added and/or adapted the probing questions accordingly.

**Problem**

“Chronically ill patients often experience psychosocial problems in everyday life. The psychosocial component of care appears to be poorly embedded in the Dutch primary care system.”

1. What is your opinion about this statement? Do you recognize this observation? Why (not)?
2. Can you explain the state-of-art of biopsychosocial care in the Dutch health care system?
3. How do you consider, from your perspective, the needs (relevance/urgency) to improve biopsychosocial care?

**Improvement of biopsychosocial care**

From your perspective (as representative of…), how do you perceive further implementation of the psychosocial component in chronic care? What steps need to be taken and by whom?

**Barriers**

From your perspective (as representative of…), what are the challenges and obstacles to successful implementation of biopsychosocial care? How can these obstacles be overcome?
